# An Unexpected Late Recurrence of Breast Irradiation-Induced Angiosarcoma Following Autologous Microvascular Breast Reconstruction

**DOI:** 10.7759/cureus.54741

**Published:** 2024-02-23

**Authors:** Mohammed Herieka, Kavit Amin, Damir Kosutic

**Affiliations:** 1 Plastic and Reconstructive Surgery, The Christie NHS Foundation Trust, Manchester, GBR

**Keywords:** sarcoma, breast cancer, radiation induced angiosarcoma, microvascular reconstruction, autologous breast reconstruction

## Abstract

The authors present the case of a 68-year-old female who developed recurrent angiosarcoma, a rare but recognized complication after breast irradiation therapy in the treatment of breast cancer. Microvascular breast reconstruction was performed after the completion of 10 years of disease-free clinical surveillance. Abdominal tissue was harvested and transferred onto the chest wall with restoration of its blood supply using microsurgical techniques to recreate the breast. Unexpectedly, local recurrence of irradiation-induced angiosarcoma was confirmed in the reconstructed breast 12 years later, a unique finding, given the long latent period and recruitment of tissues from a distant site. It is vital to consider the potential of late recurrence before embarking on complex reconstructions, and this should be discussed with patients who have a history of angiosarcoma. This further emphasizes the importance of long-term surveillance in such a rare, yet aggressive tumor at specialist centers.

## Introduction

Breast angiosarcoma arising secondary to irradiation for the treatment of primary breast carcinoma is a rare and aggressive, yet well-documented complication. Breast angiosarcoma is implicated in 8% of all angiosarcomas, and for women undergoing adjuvant radiotherapy, the risk of developing angiosarcoma is 0.05% to 0.3% [1]. Angiosarcoma has a propensity to metastasize, and unlike more commonly encountered soft tissue tumors, its behavior and pathological characteristics are ill-understood. This is primarily due to its rarity, and as a consequence, targeted treatment options remain limited. There is a paucity of clinical trials available to guide the clinical management of this condition. Treatment necessitates radical excision with wide and deep margins to mitigate the risk of local recurrence and prevent further progression into metastatic disease. However, there is currently no consensus on the precise margins of clearance required.

The national mastectomy and breast reconstruction audits have demonstrated an improvement in patient satisfaction and well-being with autologous reconstruction versus no reconstruction [2]. Following routine clinical surveillance, when deemed acceptably safe, patients may seek to have breast reconstructive surgery to restore form. The gold standard reconstructions in the modern era are based on implant or autologous techniques. While there is no firm consensus on the superiority of either approach, there is weak evidence to suggest that implant-based reconstructions are less cost-effective and perform less well in patient-reported outcome measures [3]. In reality, the choice of reconstruction depends on patient characteristics and preferences. Autologous techniques primarily involve the transfer of tissue from the abdomen [4], thigh [5], or buttock [6]. They are performed via specialist teams that require microvascular training and the use of an operative microscope.

The demonstrated potential for late recurrence of irradiation-induced breast angiosarcoma emphasizes the importance of investigating any history indicative of angiosarcoma or related symptoms when contemplating reconstruction.

## Case presentation

The patient was diagnosed with diffuse Grade II node-negative breast ductal carcinoma in 2001. She underwent a right mastectomy, axillary node clearance, and adjuvant radiotherapy. She then completed courses of adjuvant hormonal treatment (Tamoxifen) and aromatase inhibitors (Anastrazole). Six years later, she presented with a subcutaneous nodule over the medial mastectomy scar. Excision biopsy revealed a well-to-moderately differentiated angiosarcoma focally extending into the underlying pectoralis major muscle. The tumor measured 25 mm x 18 mm x 14 mm; it was 7 mm from the deep margin and 11 mm from the radial margin of the excision specimen. The adjacent dermis exhibited radiation-induced changes. Staging computed tomography (CT) showed no evidence of distant disease.

**Figure 1:**
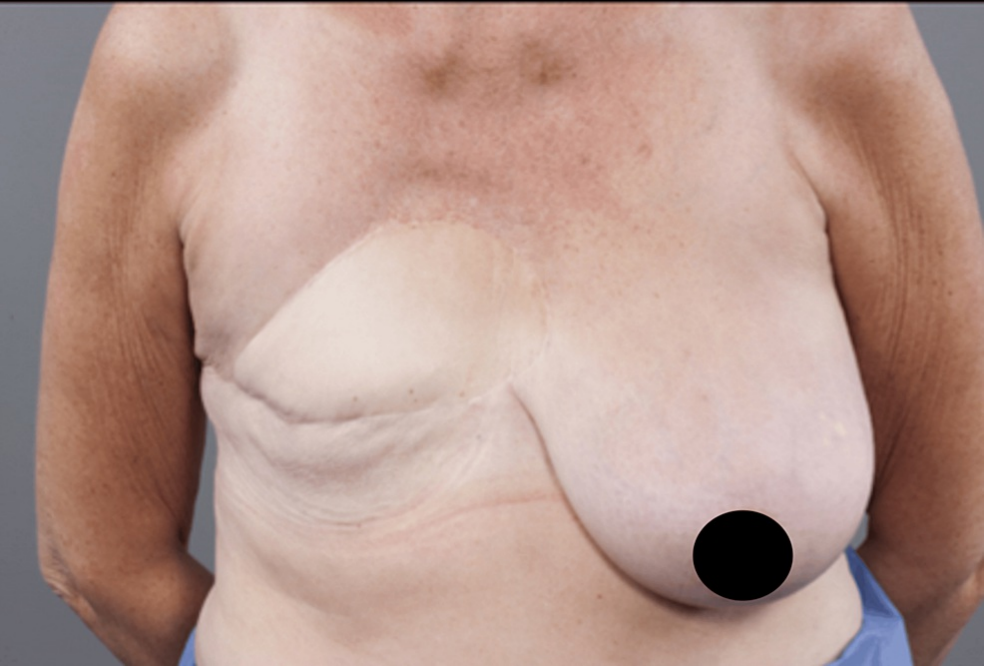
Well-healed latissimus dorsi flap after 10 years of clinical surveillance (pre-DIEP flap reconstruction) DIEP, deep inferior epigastric artery perforator

**Figure 2:**
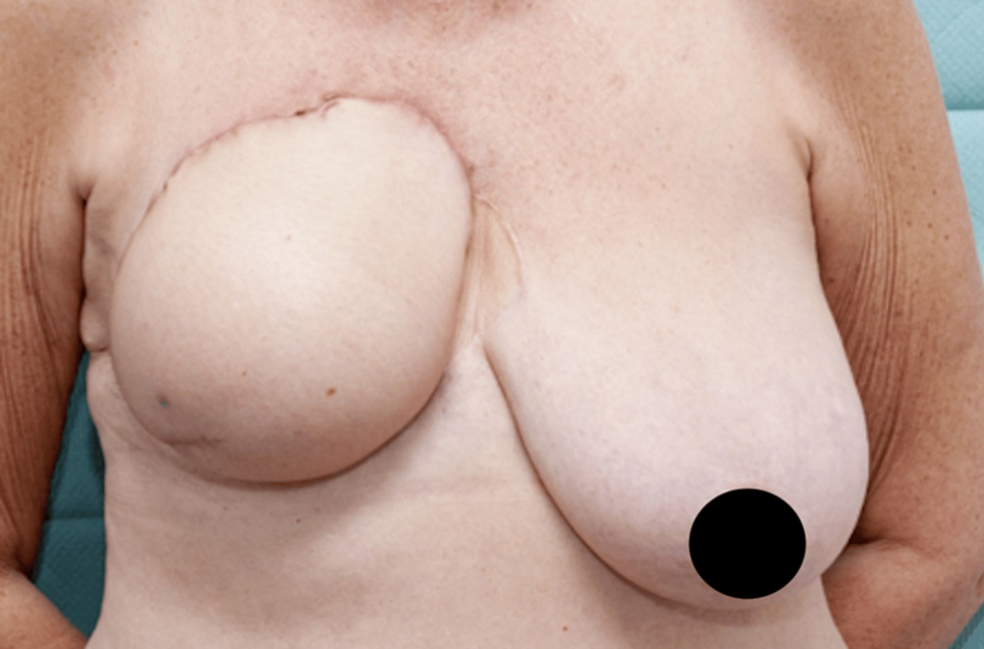
Post-DIEP flap reconstruction of the right breast. Contralateral symmetrizing mastopexy was later performed. DIEP, deep inferior epigastric artery perforator

This secondary, irradiation-induced angiosarcoma was treated through radical excision of the skin, subcutaneous fat, and pectoralis major and minor muscles, with clinical margins of 2-3 cm. The Latissimus dorsi muscle and a portion of the overlying skin and soft tissue were transferred from the back and transposed onto the chest wall to reconstruct the defect (Figure 1). The patient then completed her oncological follow-up consisting of 10 years of clinical and radiological surveillance. The patient then underwent a right breast-free deep inferior epigastric artery perforator flap (DIEP) in October 2017 to improve cosmesis (Figure 2). The abdominally based flap was anastomosed to the internal mammary and thoracodorsal arteries using an operating microscope. The DIEP flap provides both good volume and tissue match with healthy skin and fat from a distant site to the primary tumor and irradiated field. A contralateral symmetrizing mastopexy was later performed.

**Figure 3:**
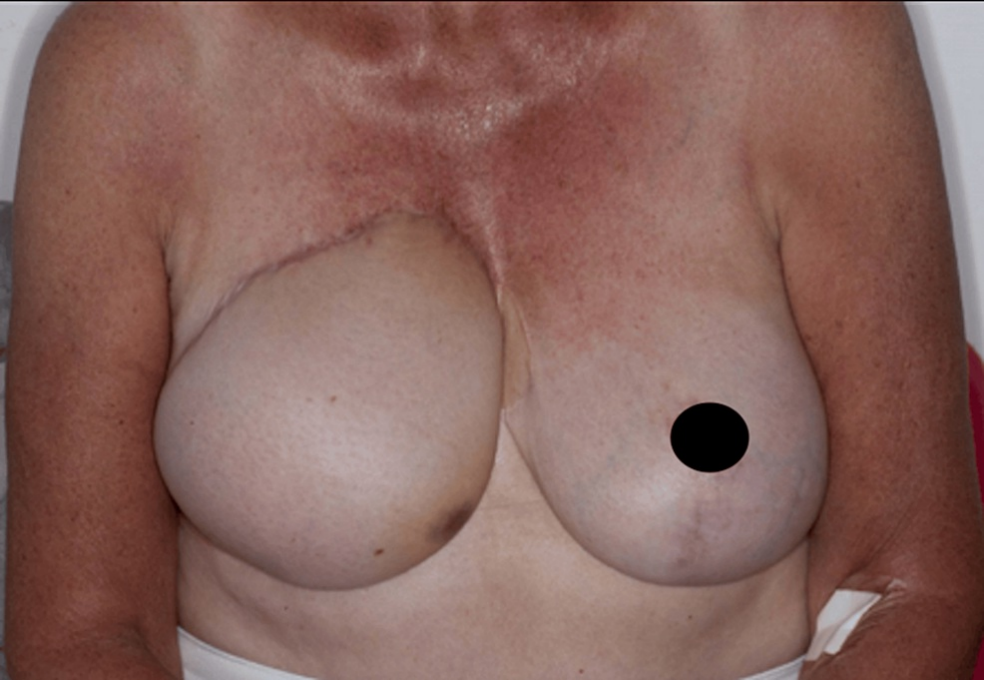
Mild ecchymosis over the inferomedial edge of the right DIEP flap breast reconstruction that was proven to be an angiosarcoma recurrence in 2017. DIEP, deep inferior epigastric artery perforator

**Figure 4:**
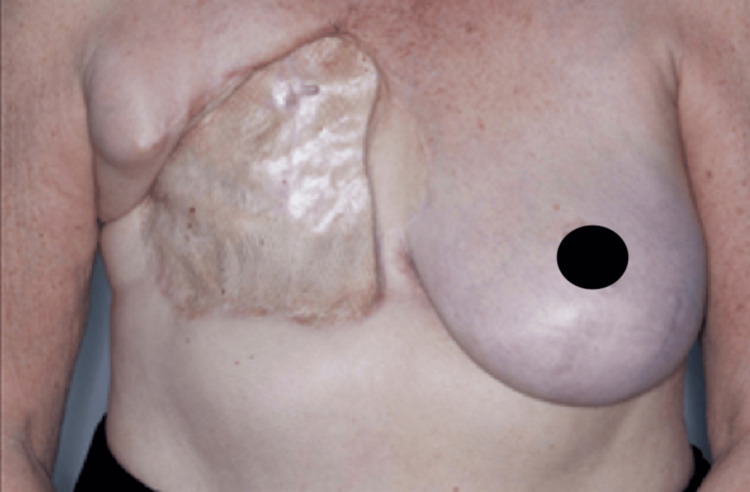
Postsurgical treatment of the angiosarcoma recurrence with extended mastectomy, removal of the DIEP flap, and skin graft reconstruction in 2017. DIEP, deep inferior epigastric artery perforator

Eleven years following the treatment of her angiosarcoma, she developed persistent ecchymosis over the inferior DIEP flap. Mapping biopsies demonstrated atypical cellular infiltration with marked nuclear pleomorphism within the dermis. Immunohistochemistry revealed strong and diffuse staining with CD31 and ERG, supporting the diagnosis of recurrent angiosarcoma (Figure 3). This was treated with excision of the entire DIEP reconstruction and split-thickness skin grafting of the underlying rib perichondrium (Figure 4).

**Figure 5:**
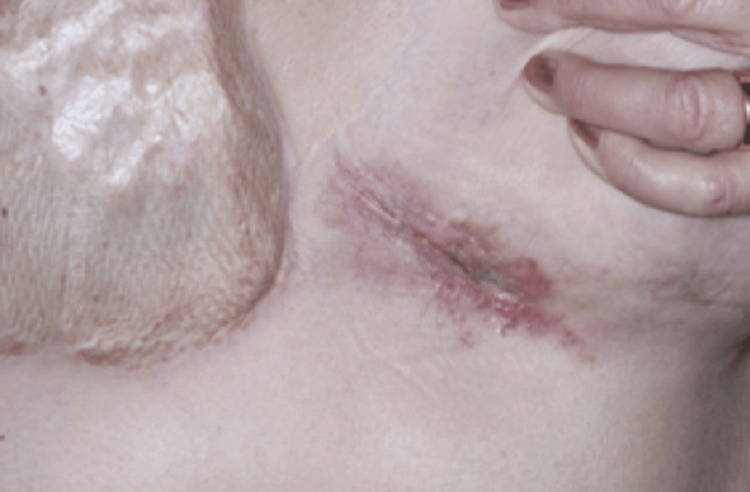
Further local recurrence in the inframammary fold of the contralateral breast in 2020.

Surprisingly, this patient developed a further chest wall recurrence, but this time on the contralateral inframammary fold in December 2020 (Figure 5). This was managed with a 2 cm margin excision and direct closure. She remained under close surveillance with no radiological evidence of metastatic disease.

## Discussion

Angiosarcoma is a rare, soft-tissue sarcoma of endothelial origin and represents 2% of all sarcomas. Its incidence has been increasing over the past 30 years largely due to radiotherapy to treat breast cancer. There is an equal distribution between genders, and they are more frequently encountered in the elderly, although they have been observed in younger age groups [7]. Most occur spontaneously; however, secondary causes are related to lymphedema and irradiation, both considered independent risk factors [7]. Breast irradiation-induced angiosarcoma has a peak incidence of 5-10 years after radiotherapy. They have a poor prognosis, with an overall five-year survival of 35%. Patient factors such as advanced age or comorbidities in addition to disease-specific factors such as the size of the tumor and metastases at presentation are postulated to reduce survival [7]. Clinicians can expect to encounter this rare and aggressive tumor more frequently owing to the increased adjuvant treatment of breast carcinomas with this radiotherapy [8]. The reason for reporting this case is to promote a high index of suspicion among clinicians and highlight the requirement for early biopsy.

Typical presentations arise with a bruise or purple papule, often resembling a benign lesion, and as such lead to a delay in diagnosis. Radiation-induced angiosarcoma affects the dermis of the breast, occasionally developing within breast parenchyma after 7-10 years [9]. Conversely, primary disease arises within the breast parenchyma and later infiltrates the skin [7]. Secondary angiosarcoma may appear as generalized skin changes, including rash, ecchymosis, bluish nodules, or skin thickening close to the site of the primary [9,10].

The histological hallmarks are abnormal, pleomorphic malignant endothelial cells. They typically express endothelial markers such as CD31, CD34, and VEGF; however, the loss of tumor differentiation can lead to the absence of these markers, making the diagnosis challenging. Secondary disease is reportedly associated with mutations in BRCA2, and the presence of these should further raise suspicion [11].

The preoperative workup is best conducted with an MRI to assess the extent of the tumor, providing valuable diagnostic information compared to mammography [7]. CT staging is also useful to exclude distant disease when radical surgery is planned, with the liver and lung the most common sites of metastases [12]. Staging is based on the American Joint Committee on Cancer (AJCC) guidance and follows the Tumor, Node, and Metastasis (TNM) system [13]. Up to 80% (50%-80%) of patients present with localized disease; however, a significant proportion (20%-45%) may have metastatic disease at presentation [14]. As with the majority of breast diagnoses, a core biopsy is recommended, as fine-needle aspiration has a high false-negative rate [15].

The evidence underpinning the management of angiosarcoma is largely the result of retrospective case series, and there is a paucity of randomized controlled trials or prospective series. These patient groups should be managed in specialized centers with an established sarcoma multidisciplinary team that has the capacity for breast reconstruction. Radical surgery with complete excision is the treatment of choice for patients presenting with localized disease. Radiotherapy has been shown to improve local control and survival in non-irradiation-induced angiosarcoma. However, radiotherapy alone is ineffective compared to its use as an adjunct to surgery [7]. Reports have demonstrated a potential role for electrochemotherapy in managing cutaneous recurrences in which radiotherapy is contraindicated. However, a short disease-free interval was observed with further recurrences detected within two months, suggesting the need for a multimodal approach [16].

Breast reconstruction significantly improves patient well-being and is typically undertaken following treatment for primary breast carcinoma. In this setting, the National Institute of Health and Care Excellence (NICE) advocates offering immediate reconstruction unless precluded by patient comorbidities [17]. Breast reconstruction practices are broadly divided into implant or autologous-only techniques. Autologous reconstruction includes pedicled (artery is not detached from its source) or free flap (detachment of source artery for anastomosis at a distant site) reconstruction. A holistic and individualized patient assessment dictates the choice between implant and autologous techniques. Reconstruction with a free (DIEP) flap remains the autologous gold standard [18]. This procedure involves surgically harvesting lower abdominal skin and fat, along with its arterial and venous supply. Subsequently, it is transferred onto the chest wall by reconnecting it to local blood vessels using a microscope, to recreate the breast. There are currently no guidelines on the timing of breast reconstruction following surgical management of breast angiosarcoma. In this case, the transfer of tissue distant from the radiotherapy field after 10 years of disease-free survival was deemed to be a safe window of oncological clearance. The European Sarcoma Network working group recommends 8-10 years of surveillance for patients with high-grade sarcoma [19]. However, even with complete surgical margins, local recurrence rates of up to 59% have been observed with a median time to recurrence of six months (range 1-78 months) [20]. The development of local recurrence within the reconstruction after a latent period of 12 years highlights the need for prospective studies to guide the management of a rare and aggressive disease.

## Conclusions

In conclusion, the management of irradiation-induced angiosarcoma demands a nuanced and vigilant approach. Our findings highlight an unexpected challenge - the recurrence of angiosarcoma within tissues transferred from a site distant to the original radiotherapy field after a disease-free period of more than 10 years. This underscores the need for consideration of potential late recurrences when counseling patients contemplating microvascular breast reconstruction. Clinicians must maintain a high degree of clinical suspicion for any skin changes within a radiotherapy field or subsequent reconstruction. Follow-up in specialized centers remains the cornerstone of the successful long-term care of these patients. These centers, equipped with multidisciplinary teams, offer a comprehensive framework for navigating the complexities associated with this rare and aggressive malignancy. 
